# A Pediatric Case of Cogan’s Syndrome With Internal Otitis

**DOI:** 10.7759/cureus.66742

**Published:** 2024-08-12

**Authors:** Yoshiyuki Sasano, Fumihiro Mochizuki, Manabu Komori

**Affiliations:** 1 Department of Otolaryngology, St. Marianna University School of Medicine, Kawasaki, JPN

**Keywords:** vemp, vhit, endolymphatic hydrops, cochlear implantation, internal otitis, cogan’s syndrome

## Abstract

Cogan’s syndrome is characterized by ocular symptoms and auditory vestibular dysfunction. Auditory vestibular dysfunction in Cogan’s syndrome is believed to be similar to Ménière’s disease, but the cause is not known in detail. We present the case of a 10-year-old boy with Cogan’s syndrome. The patient had panuveitis, bilateral hearing loss, and bilateral vestibular dysfunction. MRI revealed no evidence of endolymphatic hydrops, which is a cause of Ménière’s disease, and enhanced contrast effects on the bilateral cochlear and vestibular apparatus. The caloric test, the video-head impulse test, and the vestibular evoked muscle potential test also showed severe vestibular dysfunction. Based on the above, the auditory vestibular dysfunction in this patient was considered to be caused by internal otitis. The patient’s vision recovered after treatment with steroids and immunosuppressive drugs, but his hearing did not recover. He underwent bilateral cochlear implantation and had a good postoperative course but we encountered difficulty in deciding when to perform cochlear implantation. This case demonstrates the importance of determining the timing of surgery in consideration of the ossification and fibrosis of the inner ear and the drug administration status.

## Introduction

Cogan’s syndrome was proposed by David G. Cogan of the United States in 1955 as a syndrome with nonsyphilitic interstitial keratitis (IK) and vestibular auditory symptoms [1]. Typical symptoms include ocular symptoms such as conjunctivitis and loss of vision due to keratitis, inner ear symptoms such as dizziness and tinnitus similar to Ménière’s disease [2,3], and hearing loss progressing gradually and leading to deafness within one to two months. Cochlear implantation is a valuable rescue surgical strategy for functional maintenance in cases with severe sensorineural hearing loss unresponsive to corticosteroids and immunosuppressive regimens. This case report discusses the cause of balance dysfunction through the results of balance function tests and imaging in a pediatric patient with Cogan’s syndrome and describes the timing of cochlear implantation.

## Case presentation

A 10-year-old boy presented with a two-month history of left-sided hearing loss and dizziness. He visited the ENT department of a general hospital and was diagnosed with sudden hearing loss. Despite treatment with tapering doses of prednisone (30 mg), no improvement was observed. Subsequently, he became aware of bilateral ocular hyperemia and vision loss and was diagnosed with bilateral panuveitis after a thorough examination at the ophthalmology department of the same hospital.

Pure tone audiometry indicated severe to profound sensorineural hearing loss on the right side at 115 dB and on the left side at 100 dB using the four-frequency average method. A caloric test revealed bilateral semicircular canal paralysis. He was suspected of having an immune disorder and was referred to our hospital.

**Figure 1:**
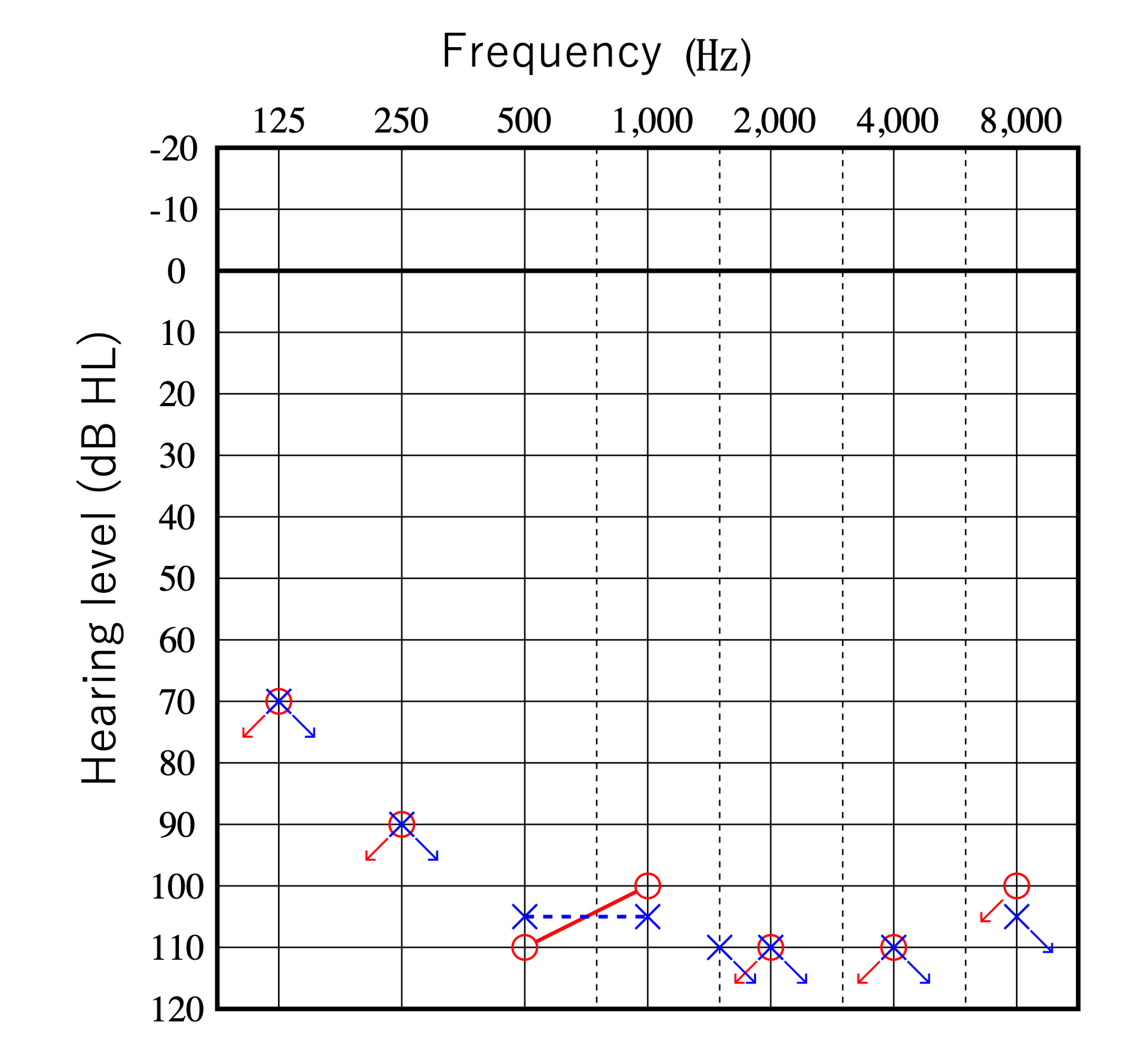
Preoperative audiograms. Right: red; left: blue.

At our clinic, audiometric tests confirmed bilateral severe to profound hearing loss (Figure 1).

**Figure 2:**
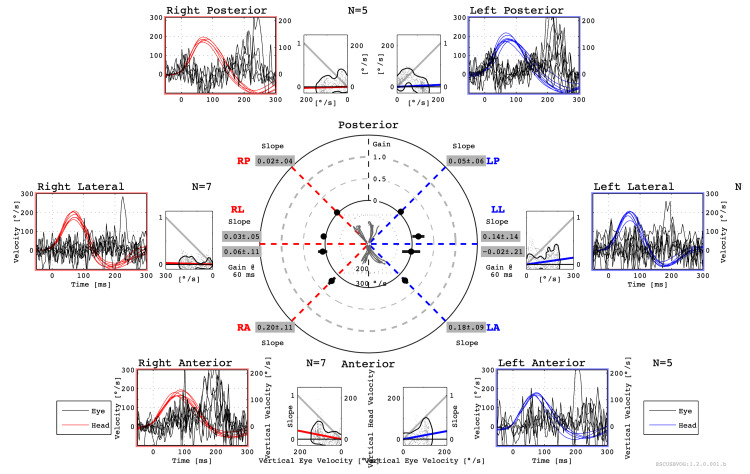
Result of vHIT. vHIT comparative superimposed head (right: red; left: blue) and eye (black) velocity in degrees/second (y-axis) vs. time in ms (x-axis). vHIT showed decreased VOR gain with corrective catch-up saccades of all semicircular canals bilaterally. vHIT: video-head impulse test; VOR vestibulo-ocular reflex

**Figure 3:**
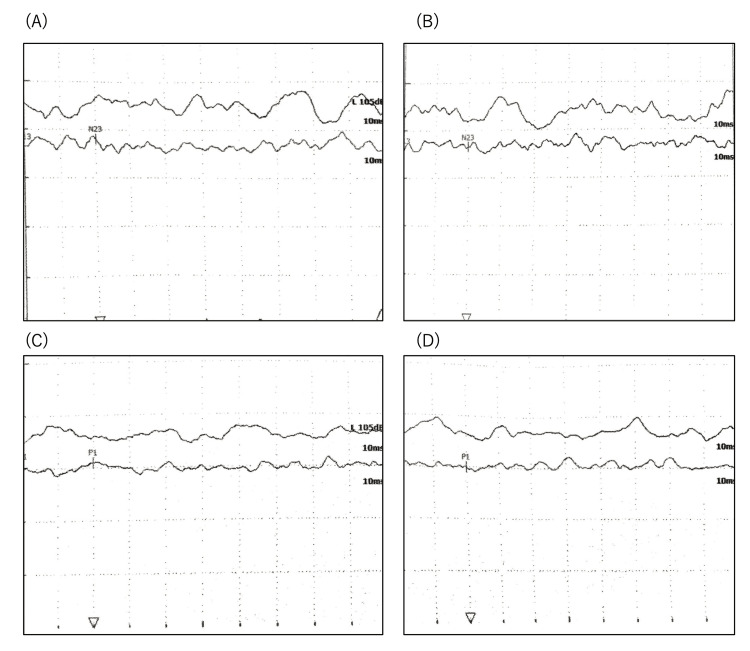
Result of VEMP. Waveforms of cVEMP (500 Hz (A: left, B: right)) and oVEMP (500 Hz (C: left, D: right)). the stimulation sounds were a tone burst of 500 Hz at 100 dBpeSPL. VEMP showed the absence of evoked potentials bilaterally. VEMP: vestibular evoked muscle potential

No nystagmus was observed. Visual acuity was 20/20 on the right and 20/40 on the left, with poor bilateral fundus translucency. The video-head impulse test (vHIT) showed decreased vestibulo-ocular reflex gain (Figure 2), and the vestibular evoked muscle potential (VEMP) test showed bilateral non-response to both cVEMP and oVEMP (Figure 3), indicating severe bilateral vestibular dysfunction.

**Figure 4:**
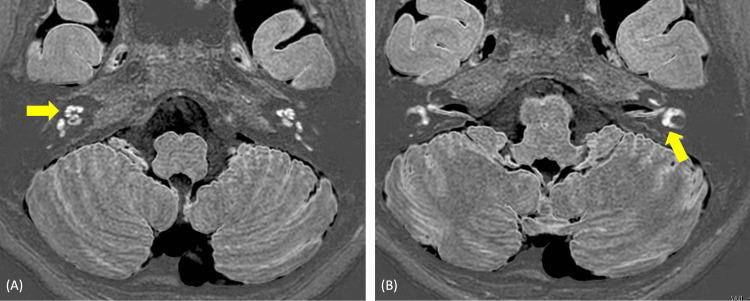
Cochlear and vestibular MRI. A: Yellow arrow indicates cochlea. B: Yellow arrow indicates vestibular. HYDROPS showed enhanced contrast effects on bilateral cochlear and vestibular.

Contrast-enhanced MRI to identify endolymphatic edema: heavily T2-weighted three-dimensional fluid-attenuated inversion recovery and HYbriD of reversed image of positive endolymph signal and native image of positive perilymph signal (HYDROPS) [4] showed enhanced contrast effects on the bilateral cochlear and vestibular without endolymphatic hydrops (Figure 4).

**Figure 5:**
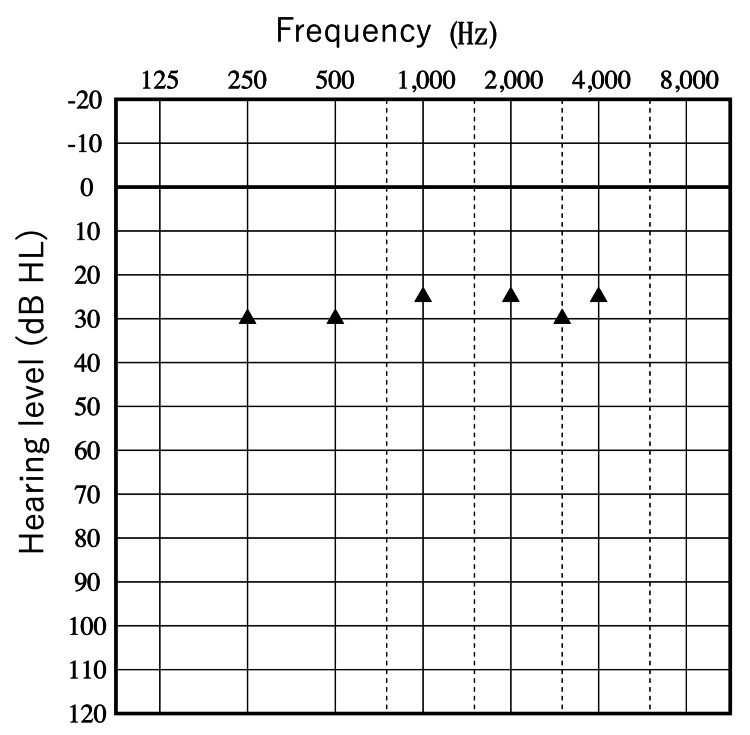
Postoperative hearing threshold in the free field.

Differential diagnoses included Blau syndrome, juvenile neurosarcoidosis, and Cogan’s syndrome. He was diagnosed with Cogan’s syndrome based on the absence of arthritis or skin symptoms. The patient received steroid pulse therapy with 1,000 mg of methylprednisolone for three days over two courses. The panuveitis resolved and his vision acuity improved to 20/16 on the right and 20/28 on the left, but his hearing did not improve. Prednisone was systematically tapered from an initial dose of 30 mg. Methotrexate was administered orally at a weekly dosage of 10 mg, while infliximab was administered intravenously at a dosage of 175 mg. The frequency of infliximab administration was gradually adjusted to biweekly and subsequently to every four weeks. Bilateral cochlear implantation was performed after confirming no ossification and fibrosis in the inner ear by imaging and when the prednisone dose was reduced to <0.3 mg/kg (12mg). It had been 80 days since the start of steroid pulse therapy until the day of surgery. At the time of surgery, methotrexate treatment was maintained, with the last dose of infliximab administered 18 days before the surgical procedure. The postoperative hearing threshold in the free field was 25-35 dB at all frequencies (Figure 5). Two and a half years after surgery, the patient had no wound infection, and the hearing threshold in the free field showed the same results as immediately after surgery. Though the patient still had a floating sensation of vertigo, he was able to walk.

## Discussion

Cogan’s syndrome is a rare disease with unknown etiology and no precise morbidity data. In 1980, Haynes et al. defined two types of Cogan’s syndrome, a typical variant and an atypical variant [2]. Typical Cogan’s syndrome is defined by (1) ocular symptoms, classically presenting as nonsyphilitic IK; (2) audio-vestibular symptoms similar to those of Ménière’s disease; and (3) an interval between the onset of ocular and audio-vestibular manifestations of <2 years. Atypical Cogan’s syndrome is characterized by (1) different inflammatory ocular manifestations, with or without IK; (2) audio-vestibular symptoms (usually progressive hearing loss); and (3) a delay of >2 years between the onset of ocular and audio-vestibular manifestations. In many cases, it is difficult to differentiate between the two types of Cogan’s syndrome because some patients do not present IK at the onset of the disease or develop this condition during the following years. In this case, the ocular and auditory symptoms developed within two years but were not accompanied by keratitis, so it is difficult to distinguish between typical and atypical types. The diagnosis of Cogan’s syndrome is mainly clinical and is based on audio-vestibular symptoms and ocular inflammation. There are no established global diagnostic criteria. The prognosis for visual acuity is relatively favorable, whereas hearing loss often remains poor, with 25-50% of cases showing no improvement [2]. In this case, the ocular symptoms and visual acuity improved with medication, but the hearing loss did not.

As mentioned above, auditory vestibular dysfunction associated with Cogan’s syndrome is often reported to resemble Ménière’s disease and is thought to result from endolymphatic hydrops. Examinations to diagnose inner-ear endolymphatic hydrops include the glycerol test, furosemide-loaded VEMP, and electrocochleography. In a previous report, a glycerol test and cochocardiogram showed endolymphatic hydrops in Cogan’s syndrome [5]. However, due to the bilateral severe auditory vestibular dysfunction detected in this case, the performance of these tests proved to be challenging. HYDROPS emerges as a valuable modality for assessing endolymphatic hydrops in such individuals. HYDROPS showed enhanced contrast effects on the bilateral cochlear, vestibular, and semicircular canals without endolymphatic hydrops. The caloric test, vHIT, and VEMP also showed vestibular hypofunction. Consequently, the auditory vestibular dysfunction of this case was attributed to internal otitis rather than endolymphatic hydrops.

Treatment of Cogan’s syndrome involves long-term administration of immunosuppressive drugs and prednisone. Cochlear implantation was performed when these drugs did not improve hearing loss. The timing of cochlear implantation poses a challenge, similar to other autoimmune conditions where surgical intervention should be delayed until the inflammatory manifestations are under medical control. The timing of cochlear implantation should be carefully deliberated, considering the risks of postoperative infection and ossification or fibrosis of the inner ear. Regarding ossification and fibrosis of the inner ear in Cogan’s syndrome, it was reported that early cochlear ossification and fibrosis can develop as early as eight weeks after the onset of deafness and is detected as early as eight weeks after the onset of hearing loss [6]. On the other hand, the risk of postoperative infection involving the insertion of artificial objects has often been reported in the field of orthopedics. The relative risk of postoperative infection in hip or knee arthroplasty is reported to be 2.03 times higher in steroid users of 1-15 mg/day and 23.1 times higher in users of high-dose steroids (>15 mg/day) [7]. This report showed the administration of non-biologic disease-modifying anti-rheumatic drugs (including methotrexate) and biologics agents (including infliximab) as not increasing the risk of infection [7]. However, there is a reported four-fold increased risk of serious infections in patients receiving tumor necrosis factor-alpha inhibitors [8]. In this case, cochlear implantation was performed after consultation with the pediatrician, immunosuppressive agents were continued, and the prednisone dose was reduced to <0.3 mg/kg/day (12 mg). When deciding on the timing of surgery, it is important to consider the drug administration status and to confirm the absence of ossification or fibrosis of the inner ear by CT or MRI.

This is a case report of a single case, and further studies with multiple cases and long-term outcomes are needed for the generalization of vestibular function assessment, timing of cochlear implantation, and long-term evaluation.

## Conclusions

This case report provides valuable insights into the management of Cogan’s syndrome in a pediatric patient, particularly regarding auditory vestibular dysfunction and cochlear implantation. We discussed the results of vestibular function tests and imaging studies, along with the decision on the timing of surgery for cochlear implantation. While it is a significant contribution to understanding this rare condition, further research involving multiple cases and long-term outcomes is needed to validate and generalize the findings.
